# Hydroxy-α-sanshool isolated from *Zanthoxylum bungeanum* Maxim. has antidiabetic effects on high-fat-fed and streptozotocin-treated mice *via* increasing glycogen synthesis by regulation of PI3K/Akt/GSK-3β/GS signaling

**DOI:** 10.3389/fphar.2022.1089558

**Published:** 2022-12-13

**Authors:** Qing Zhang, Ruo-Lan Li, Ling-Yu Wang, Ting Zhang, Die Qian, Dan-Dan Tang, Cheng-Xun He, Chun-Jie Wu, Li Ai

**Affiliations:** ^1^ State Key Laboratory of Southwestern Chinese Medicine Resources, School of Pharmacy, Chengdu University of Traditional Chinese Medicine, Chengdu, China; ^2^ Innovative Institute of Chinese Medicine and Pharmacy, Academy for Interdiscipline, Chengdu University of Traditional Chinese Medicine, Chengdu, China; ^3^ School of Ethnic Medicine, Chengdu University of Traditional Chinese Medicine, Chengdu, China

**Keywords:** type 2 diabetes mellitus, hydroxy-α-sanshool, *Zanthoxylum bungeanum* Maxim., glycogen synthesis, PI3K/Akt/GSK-3β pathway

## Abstract

Type 2 diabetes mellitus (T2DM) is a chronic metabolic disease characterized by hyperglycemia. The fruits of *Zanthoxylum bungeanum* Maxim. is a common spice and herbal medicine in China, and hydroxy-α-sanshool (HAS) is the most abundant amide in *Z. bungeanum* and reported to have significant hypoglycemic effects. The purpose of this study was to evaluate the ameliorative effects of HAS on T2DM and the potential mechanisms responsible for those effects. An acute toxicity test revealed the median lethal dose (LD50) of HAS is 73 mg/kg. C57BL/6 J mice were fed a high-fat diet and given an intraperitoneal injection of streptozotocin (STZ) to induce T2DM in mice to evaluate the hypoglycemic effects of HAS. The results showed that HAS significantly reduced fasting blood glucose, reduced pathological changes in the liver and pancreas, and increased liver glycogen content. In addition, glucosamine (GlcN)-induced HepG2 cells were used to establish an insulin resistance cell model and explore the molecular mechanisms of HAS activity. The results demonstrated that HAS significantly increases glucose uptake and glycogen synthesis in HepG2 cells and activates the PI3K/Akt pathway in GlcN-induced cells, as well as increases GSK-3β phosphorylation, suppresses phosphorylation of glycogen synthase (GS) and increases glycogen synthesis in liver cells. Furthermore, these effects of HAS were blocked by the PI3K inhibitor LY294002. The results of our study suggest that HAS reduces hepatic insulin resistance and increases hepatic glycogen synthesis by activating the PI3K/Akt/GSK-3β/GS signaling pathway.

## Introduction

Increasing epidemiological evidence has revealed that type 2 diabetes mellitus (T2DM) is emerging as a considerable socioeconomic pressure on individuals and society due to the huge cost of associated healthcare (Yu et al., 2015). Patients with T2DM commonly have insulin deficiency due to insulin resistance and pancreatic β-cell dysfunction (Vishvanath and Gupta, 2019; Blahova et al., 2021). Insulin resistance is considered the predominant contributing factor in T2DM, which is mainly caused by obesity, sedentary lifestyle, and increasing life span (DeFronzo and Tripathy, 2009). In addition, T2DM is often accompanied by many complications, such as cardiovascular, kidney, and retinal diseases, which are becoming a serious challenge to the improvement and maintenance of human health. Currently, metformin is the first-line drug for treating T2DM. Other available drugs include α-glucosidase inhibitors (such as acarbose), sulfonylureas (such as glimepiride), and DPP-4 inhibitors (such as sitagliptin). However, all available drugs have serious side effects, such as renal impairment, gastrointestinal disorders, and hypoglycemia, among others (Deng et al., 2017). Therefore, the identification of more candidate antidiabetic monomers from natural herbal medicines, with relatively fewer and less serious side effects, will be beneficial in the prevention and clinical treatment of T2DM.

The fruit of *Zanthoxylum bungeanum* Maxim. (Rutaceae family) has been a popular spice and herbal medicine in China for thousands of years. Hydroxy-α-sanshool (C_16_H_25_NO_2_, HAS) is the primary active amide isolated from the fruit of *Z. bungeanum*, which has versatile bioactivities, such as anti-Alzheimer’s disease, anti-obesity, and lipid-lowering effects (Wang et al., 2019; Li et al., 2020). In addition, previous studies have reported that amides in the fruit of *Z. bungeanum* have potential hypoglycemic effects (You et al., 2015; Ren et al., 2017a; Ren et al., 2017b). As part of our continuing work in exploring the medical value of the fruit of *Z. bungeanum*, we evaluated the antidiabetic effects of HAS on high-fat-fed and streptozotocin-treated mice, and further explored the associated molecular mechanisms. Our study results suggest that the development of HAS as a clinical treatment for T2DM would be beneficial.

## Materials and methods

### Animals

Male C57BL/6 J mice (20 ± 2 g) and ICR mice (20 ± 2 g) were obtained from Si Pei Fu Biotech (http://www.spf-tsinghua.com/, Beijing, China). This study was carried out following international guidelines for animal experiments and approved by the Animal Care and Use Committee of Chengdu University of Traditional Chinese Medicine (Ethical approval: No. 2020-27). The animals were adaptively reared in an SPF-grade environment for 1 week on a 12 h light/12 h dark cycle. The indoor temperature was 22°C and the relative humidity was 50%–65%. Animals were allowed to eat and drink freely during this period.

### Cells

The human hepatocellular cancer cell line HepG2 was purchased from the Guangzhou Genio Biological Co., Ltd. (Guangzhou, China, https://www.jennio-bio.com/). Cells were cultured in Dulbecco’s Modified Eagle’s Medium (DMEM), a high sugar medium containing 10% FBS, at 37°C in a humidified, 5% CO_2_ atmosphere.

### Chemicals and reagents

High-fat diet (HFD, NutriPhenomics®) was supplied by Research Diets, Inc. (New Brunswick, NJ, United States). Blood glucose meters and test strips were obtained from Roche (Shanghai, China). Serum ALT, AST, TC, and TG biochemical assay kits were purchased from Mindray Bio-Medical Electronics Co., Ltd. (Shenzhen, China). Glycated serum protein (GSP) detection kits were obtained from Shanghai Enzyme Linked Biotech (Shanghai, China). Insulin detection kits were obtained from Shenzhen Highcreation Technology Co., Ltd. (Shenzhen, China). Catalase (CAT), malonaldehyde (MDA), superoxide dismutase (SOD), and reduced glutathione (GSH) detection kits were purchased from Suzhou Michy Biomedical Technology Co., Ltd. (Suzhou, China). Glycogen detection kits were obtained from Suzhou Comin Biotechnology Co., Ltd. (Suzhou, China), and the PAS staining kits were purchased from Beijing Solarbio Technology Co., Ltd. (Beijing, China). Streptozotocin (STZ) and 2-NBDG were obtained from Shanghai Maokang Biotechnology Co., Ltd. (Shanghai, China). The EdU staining kit was purchased from Sangon Biotech Co., Ltd. (Shanghai, China). Glucosamine was purchased from Beyotime Biotechnology (Shanghai, China). LY294002 was purchased from MedChemExpress (Shanghai, China). Enhanced chemiluminescence (ECL) reagent was obtained from Beijing 4A Biotech Co. (Beijing, China). Primary antibodies for glycogen synthase (GS), phosphorylated (p)-GS, p-GSK-3β, and β-tubulin were obtained from Abmart Medical Technology Co., Ltd. (Shanghai, China). Primary antibodies for p-PI3K, PI3K, p-Akt, and Akt were purchased from Abclonal Biotechnology Co., Ltd. (Wuhan, China). The GSK-3β primary antibody was supplied from Santa Cruz Biotechnology, Inc. (CA, United States).

### Extraction and isolation

The fruit of *Z. bungeanum* was collected from Hanyuan, Sichuan Province, China in August 2020, and identification was confirmed by Prof. Chunjie Wu (School of Pharmacy, CDUTCM). A voucher specimen (#20200822) was deposited in our laboratory. HAS was extracted from the fruit following our previously established method (Zhang et al., 2019). Briefly, the fruit of *Z. bungeanum* (50 g) was crushed into powder and extracted three times, using methanol (1000 ml) and an ultrasonic extractor, for 60 min each time at 40°C. The extracts were then filtered and the filtrates were dried *via* vacuum evaporation. Next, the extract was placed in water and extracted further with ethyl acetate (EtOAc) to obtain the EtOAc fraction. The EtOAc fraction was subjected to repeated silica gel (200–300 mesh) column chromatography and eluted with petroleum ether–EtOAc (v/v, 2:1–1:2). Combination of similar fractions based on TLC analysis resulted in three fractions (I-III). By using prepared high-performance liquid chromatography (HPLC), crude HAS was isolated from fraction II. After recrystallization with complex solvents (EtOAC: n-hexane, v/v, 1:1), HAS (200 mg) with a purity of over 98% was obtained. The identification of HAS was confirmed by comparing its HPLC chromatographic analysis data with a standard reference sample of HAS (purity ≥ 98%, PUSH Bio-Technology, Chengdu, China).

### HAS Toxicity evaluation

For the acute toxicity test, 60 ICR mice were randomly divided into six groups (*n* = 10). Mice from each group were given an oral dose of HAS at 40.96, 51.20, 64.00, 80.00, 100.00, or 125.00 mg/kg, respectively. The mortality rates of the mice within a 24 h period were recorded, and the median lethal dose (LD50) value was calculated using the Bliss method.

### Type 2 diabetic mouse model and HAS treatment

Male C57BL/6 J mice (20 ± 2 g) were prepared as a model of T2DM. After adaptive feeding for 1 week, except for the mice in the normal control (NC) and NC + HAS (5 mg/kg) groups (*n* = 10), mice were administered HFD. After 1 month of continued feeding, mice in the HFD group were intraperitoneally injected with 40 mg/kg/d STZ (prepared in citrate buffer, pH 4.0) for 7 days, and the mice that were fed a normal diet received the same procedure but with the injection of citrate buffer only. The HFD + STZ mice with fasting blood glucose (FBG) levels higher than 11.1 mmol/L were then selected for the following experiments. The mice were randomly divided into five groups, including HFD/STZ (HFD feeding plus STZ treatment), HFD/STZ + Metformin (Met 150 mg/kg), HFD/STZ + HAS-L (HAS 1.25 mg/kg), HFD/STZ + HAS-M (HAS 2.5 mg/kg), and HFD/STZ + HAS-H (HAS 5 mg/kg), with 10 mice in each experimental group (Gao et al., 2016). Subsequently, HAS was dissolved in 0.5% CMC-Na, and mice in the NC + HAS (5 mg/kg) and HFD/STZ + HAS (1.25, 2.5, 5 mg/kg) groups were administered HAS orally at doses of 5 mg/kg, 1.25 mg/kg, 2.5 mg/kg, or 5 mg/kg for 4 weeks, respectively. The positive control treatment used in this study was Met, which was administered daily by gavage at 150 mg/kg. The mice in the NC and HFD/STZ groups received vehicle solvent. During the experiment, changes in blood glucose, body weight, feed intake, and water intake were recorded.

After 4 weeks of treatment, FBG was assessed by tail vein blood sampling (Roche et al., 2020) after 12 h of fasting. Following body weight measurement, blood samples were collected from the mice by orbital blood sampling. Blood samples were processed by centrifugation at 3000 rpm for 10 min, and serum was separated and stored at -80°C for subsequent biochemical analysis. Changes in liver morphology and color were noted, and weight was recorded. The liver and pancreas were then fixed and frozen for subsequent analysis.

### Oral glucose tolerance test

One week before the end of the experiment, mice were fasted without water for 12 h. Mice in each group received an intragastric glucose solution (2 g/kg) to initiate an oral glucose tolerance test (OGTT). Blood glucose was measured by tail tip blood sampling at 0, 30, 60, and 120 min after injection, and the area under the blood glucose curve (AUC) was calculated.

### Insulin tolerance tests

One week before the end of the experiment, mice were fasted without water for 12 h, and intraperitoneal injection of 0.75 U/kg insulin was performed to initiate an insulin tolerance test (ITT). Blood glucose was measured by tail tip blood sampling at 0, 30, 60, and 120 min after injection, and the AUC was calculated.

### Serum biochemical tests

The levels of alanine transaminase (ALT), glutamic-oxalacetic transaminase (AST), triglyceride (TG), and total cholesterol (TC) in the serum of mice were detected using an automatic biochemical instrument system. The levels of insulin and GSP in serum were detected according to kit instructions.

### Histopathological examinations

Liver and pancreas tissues were fixed in 10% neutral buffered formalin and subsequently embedded in paraffin, sectioned at 5 μm, de-paraffinized, and stained with hematoxylin-eosin (H and E). In addition, PAS staining was carried out to assess liver glycogen. Finally, histopathological changes and the location and abundance of glycogen in liver tissues were observed using an optical microscope.

### Determination of the oxidative stress indices in liver

Frozen liver tissue was ground with a cryogenic tissue grinder. Supernatant was collected by centrifugation and the protein concentration of the liver tissue fluid was detected using a BCA kit. The levels of SOD, CAT, MDA, and GSH in the liver were detected according to commercial kit instructions.

### Determination of glycogen levels in the liver

Frozen liver tissue was ground with a cryogenic tissue grinder. The supernatant was collected and the glycogen content in liver tissue was determined according to the instructions of the tissue glycogen detection kit.

### Cell culture and treatment

HepG2 cells were seeded into 96-well plates (1 × 10^4^ cells/well). After cell adherence, different concentrations of HAS (0–60 μg/ml) were added to the culture, and the cells were incubated for another 24 h. The CCK-8 kit was then used to detect cell viability. The cell survival rate after HAS treatment was calculated to determine the drug action concentration. Glucosamine was used to induce insulin resistance, as described previously (Sun et al., 2007). Briefly, HepG2 cells were incubated with 18 mM glucosamine (GlcN) in serum-free medium for 18 h to induce insulin resistance, followed by treatment with 2.5, 5, or 10 μg/ml HAS for 24 h. At the end of the treatment period, the CCK-8 kit was used to detect the survival rate of cells in each group. The PI3K/Akt inhibitor LY294002 (10 μM) and 10 μg/ml HAS were also used as a treatment for some cells as part of the experiment.

### EdU staining

HepG2 cells were treated with 18 mM GlcN for 18 h. HepG2 cells were then incubated with 2.5, 5, or 10 μg/ml HAS for 24 h in the presence of GlcN. The cells were subsequently incubated with EdU staining solution for 2.5 h in the dark. Next, the cell samples were fixed using 4% paraformaldehyde, and cell nuclei were stained with Hoechst dye. The EdU-positive cells were then observed and photographed using a Leica SP8SR confocal laser microscope (Wetzlar, Germany).

### Glycogen determination in HepG2 cells

After HAS treatment, the glycogen in the cells was stained *via* PAS staining solution and photographed. In addition, glycogen level was measured using a glycogen detection kit.

### Glucose intake

The fluorescent labeled analogue of 2-deoxyglucose, 2-NBDG, can be used as a tracer to evaluate cellular glycogen metabolism and simulate glucose uptake by living cells. After drug treatment, the cells were starved for 12 h and then incubated with 2-NBDG (100 uM) for 30 min; the fluorescence intensity was observed or detected by laser confocal microscopy or flow cytometry.

### Immunofluorescence assay

HepG2 cells were seeded into small glass-bottom dishes and received treatment with HAS. After HAS treatment, the cells were incubated with serum blocking solution and 0.3% Triton x-100 for 1 h at room temperature. The corresponding antibodies (GS, 1:300; p-GS, 1:300; GSK-3β, 1:300; p-GSK-3β, 1:300) were then added to the small dishes and incubated overnight at 4°C. The next day, the primary antibody was discarded, the corresponding fluorescent secondary antibody (1:500) was added, and incubation was continued for 1 h at room temperature. After incubation, cellular fluorescence was observed using a laser confocal microscope.

### Western blotting

After HAS treatment, cells were harvested, total protein was extracted, and protein concentration was determined using a BCA kit. Denaturing reagents were subsequently added to the protein and samples were heated in preparation for western blotting (WB) experiments. The proteins were then fractionated by SDS/PAGE and transferred to a PVDF membrane. The PVDF membrane was incubated in blocking solution for 1 h and then incubated overnight with the corresponding primary antibody (GS, 1:800; p-GS, 1:800; GSK-3β, 1:800; p-GSK-3β, 1:800; AKT, 1:800; p-AKT, 1:800; PI3K, 1:800; p-PI3K, 1:800) at 4°C. The next day, the PVDF membrane was incubated with horseradish peroxidase-labeled secondary antibody (1:1000) for 1 h. Finally, an ECL solution was used to detect the target protein on the PVDF membrane. Expression of the target protein was normalized using β-tubulin as an internal reference standard, and the expression level of the target protein was calculated relative to β-tubulin expression.

### Statistical analysis

Results were expressed as mean ± SD and the two-tailed Student’s t-test, with a significance level of *p* < 0.05, was used in the statistical analysis of study data.

## Results

### HAS has a promising safety profile

We investigated the possible toxic effects of HAS. As shown in Table 1, we evaluated the acute toxicity of HAS *via* assessment of the median lethal dose (LD50). The LD50 value of orally administered HAS was 73 mg/kg. In addition, our results suggested HAS had an even better safety profile at doses less than 40.96 mg/kg. In our subsequent experiments, we used orally administered HAS at doses of 1.25, 2.50, and 5.00 mg/kg, which were less than 1/10 of the LD50 value.

**TABLE 1:** LD50 value of HAS in mice.

	Dose (mg/kg)	Logarithmic dose	*n*	Death number	Death ratio (%)
1	125.00	2.097	10	10	100
2	100.00	2.000	10	9	90
3	80.00	1.903	10	6	60
4	64.00	1.806	10	3	30
5	51.20	1.709	10	1	10
6	40.96	1.612	10	0	0

LD50 = 73 mg/kg, 95% confidence interval 64.9–82.2 mg/kg.

### HAS reduces body weight and blood glucose in T2DM mice

**FIGURE 1:**
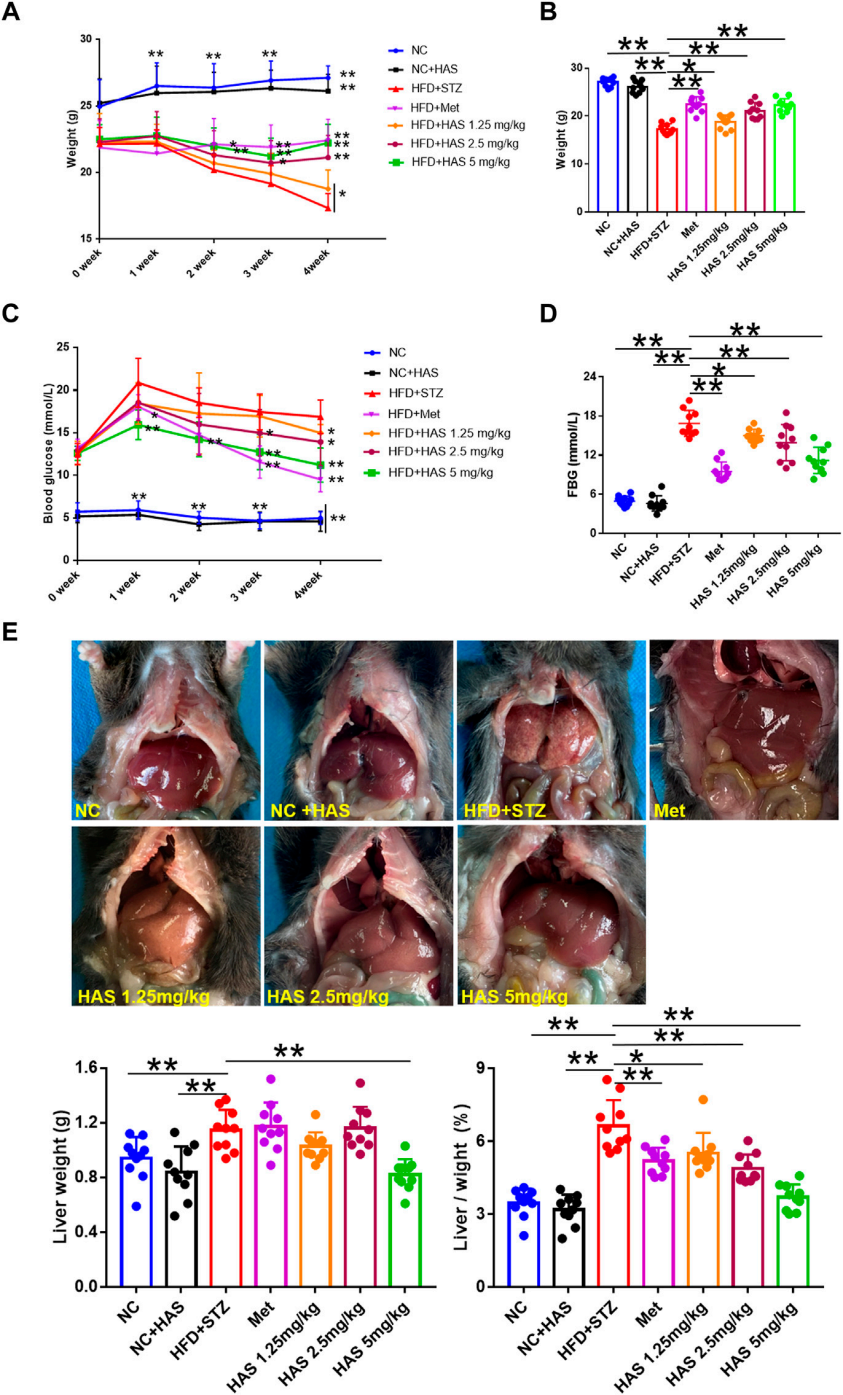
Effects of HAS on HFD + STZ mice. **(A,B)** HAS treatment resulted in increased body weight of HFD + STZ mice. **(C,D)** HAS treatment resulted in decreased fasting blood glucose in HFD + STZ mice. **(E)** Representative image of the liver of mice in different treatment groups; HAS treatment reduced liver weight and liver index in HFD + STZ mice. The doses of 2.5, 5.0, and 10 mg/kg indicate low, middle, and high doses of HAS, respectively, and the dose of Met was 150 mg/kg. Data are expressed as mean ± SD (*n* = 10), **p < 0.01 vs. HFD + STZ group.

As shown in Figure 1A, changes in body weight were recorded during the experiment. Although HFD led to weight gain, the body weight of mice decreased significantly following continuous STZ injection. During the 4 weeks, mice in the HFD + STZ groups (T2DM mice) continued to lose weight, a characteristic feature of T2DM. Fortunately, weight loss in T2DM mice could be attenuated by HAS treatment. We found that, similar to the effects of Met treatment, the body weight of HAS-treated T2DM mice (HFD/STZ + HAS-treatment) was significantly higher than that of T2DM mice (Figure 1B). In addition, results shown in Supplementary Figure S1 also suggest that HAS treatments decrease water intake (Supplementary Figure S1A) and food intake (Supplementary Figure S1B) of T2DM mice. The blood glucose of the mice was also monitored during the observation period. After continuous treatment with HFD/STZ, the FBG of T2DM mice increased sharply in the first week and then decreased gradually from 2 weeks to 4 weeks. Importantly, like Met, HAS treatment decreased FBG in T2DM mice at all observation time points (Figure 1C). In fact, after 1 week of treatment with a high dose of HAS, the FBG of the T2DM mice decreased significantly (*p* ≤ 0.01 vs. HFD + STZ, Supplementary Figure S1C). At week 3, 2.5 mg/kg HAS treatment also significantly reduced FBG in T2DM mice (Supplementary Figure S1C). At week 4, all HAS-treated groups showed significant improvement in FBG compared with the HFD + STZ group (Figure 1D). Furthermore, pathological changes in liver tissues from T2DM mice were observed. Liver tissue from normal mice was smooth and dark red in color, whereas the liver from the T2DM mice was rough and pale or pink in color. Additionally, the liver index of T2DM mice was significantly increased compared to that of the normal mice (Figure 1E). Interestingly, HAS treatment significantly attenuated the pathological changes induced by HFD + STZ (Figure 1E).

### HAS improves liver function and reduces insulin resistance in T2DM mice

**FIGURE 2:**
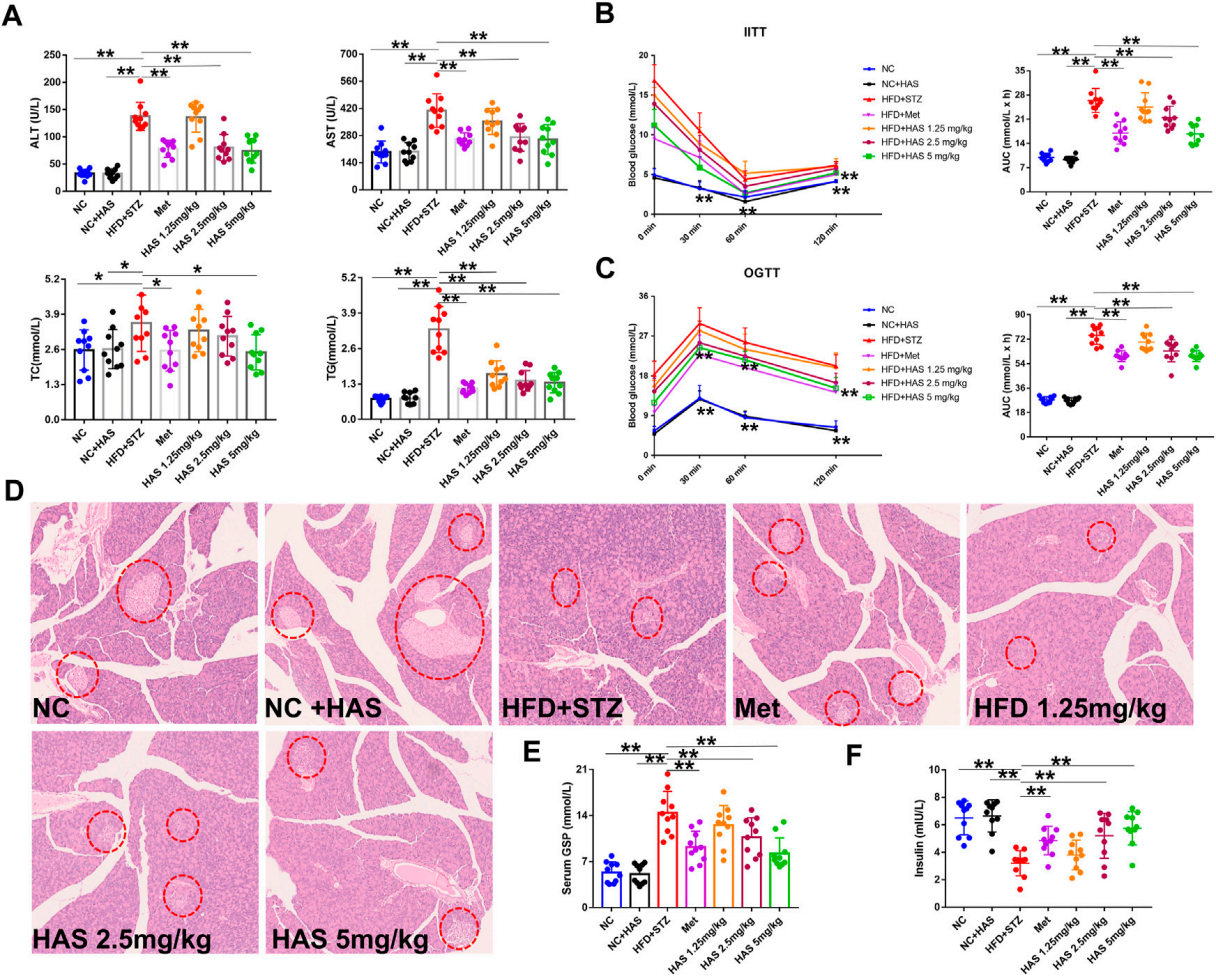
Effects of HAS on serum biochemistry and insulin resistance in T2DM mice. **(A)** Effects of HAS on ALT, AST, TC, and TG in T2DM mice. **(B)** Effects of HAS on ITT in T2DM mice. **(C)** Effects of HAS on OGTT in T2DM mice. **(D)** Effects of HAS on histopathology of the pancreas of T2DM mice; islet B cells in pancreatic tissue are marked with red circles. **(E,F)** Effects of HAS on GSP and insulin content in T2DM mice. The doses of 2.5, 5.0, and 10 mg/kg indicate low, middle, and high doses of HAS, respectively, and the dose of Met was 150 mg/kg. Data are expressed as mean ± SD (*n* = 10), **p < 0.01 vs. HFD + STZ group.

As shown in Figure 2A, compared with the NC mice, no obvious difference was observed in ALT or AST in NC + HAS treated mice, suggesting HAS has no effect on the liver function of normal mice. However, serum ALT and AST in T2DM mice were significantly increased, indicating HFD + STZ treatment caused serious disruption of liver function. Fortunately, like Met, HAS treatment reduced the levels of ALT and AST of T2DM mice, suggesting HAS could reduce liver injury of T2DM mice induced by HFD + STZ. In addition, like Met, HAS also reduced the serum levels of TG and TC of T2DM mice, indicating HAS has the capacity to improve the lipid levels of T2DM mice.

Glucose tolerance and insulin tolerance of HAS-treated mice were measured by OGTT and ITT, respectively. In the ITT experiment, the blood glucose of the NC group and NC + HAS mice decreased gradually after the intraperitoneal injection of insulin. At 60 min, the blood glucose of the mice decreased to the lowest level, then gradually increased, and recovered to the baseline level within 120 min. Similar results were observed in the other treatment groups, but none returned to baseline within 120 min. Statistical analysis of the AUC of blood glucose changes showed that STZ treatment induced a significant increase in AUC in the ITT compared with NC and NC + HFD, while both HAS and Met treatment reduced that increase in AUC (Figure 2B). The OGTT assesses the function of islet β cells and reflects the body’s ability to regulate blood sugar. As shown in Figure 2C, after gavage of glucose, the blood glucose of normal mice reached the maximum value after 30 min, and returned to the baseline blood glucose level within 120 min T2DM mice experienced a rapid increase in blood glucose after receiving glucose gavage, and the blood glucose reached its peak at 30 min, followed by a gradual decrease in blood glucose. However, the blood glucose did not fully recover within 120 min, indicating STZ + HFD treatment causes dysfunction in insulin secretion, blood glucose regulation, and glucose tolerance (Figure 2C). In addition, at the end of the experiment, the serum insulin content of T2DM mice was significantly lower than that of normal mice, indicating treatment with STZ + HFD reduced insulin secretion in mice. However, HAS and Met treatment significantly increased insulin levels in T2DM mice (Figure 2E). Similarly, the serum GSP content of T2DM mice increased sharply at the end of the experiment, suggesting blood glucose was at a high level in the most recent 1–2 weeks, which was consistent with the results of blood glucose detection. Interestingly, like Met, HAS reduced GSP levels in T2DM mice at the end of the experiment (Figure 2F). Compared with T2DM mice without HAS treatment, HAS-treated T2DM mice had significantly improved islet function and glucose tolerance. Pathological examination of the pancreas was also carried out. The pancreas of normal mice was soft and grayish red in color, and islet cells were round or oval and distributed in the pancreatic lobules, showing complete cell structure, regular arrangement, clear nucleoli, and no swelling or congestion. After treatment with HFD + STZ, the pancreas of T2DM mice appeared yellow and white in color, and the islet cells showed structural atrophy, and were small in size, reduced in number, loose in distribution, and irregular in shape, suggesting that HFD + STZ treatment damaged the pancreatic tissue. Compared with T2DM mice without HAS treatment, HAS treatment (like Met) significantly improved the physical aspects of the pancreas of T2DM mice as demonstrated by an increased number of islet cells, clearer structure, and reduced islet atrophy (Figure 2D).

### HAS treatment reduced pathological changes and oxidative stress in the liver of T2DM mice

**FIGURE 3:**
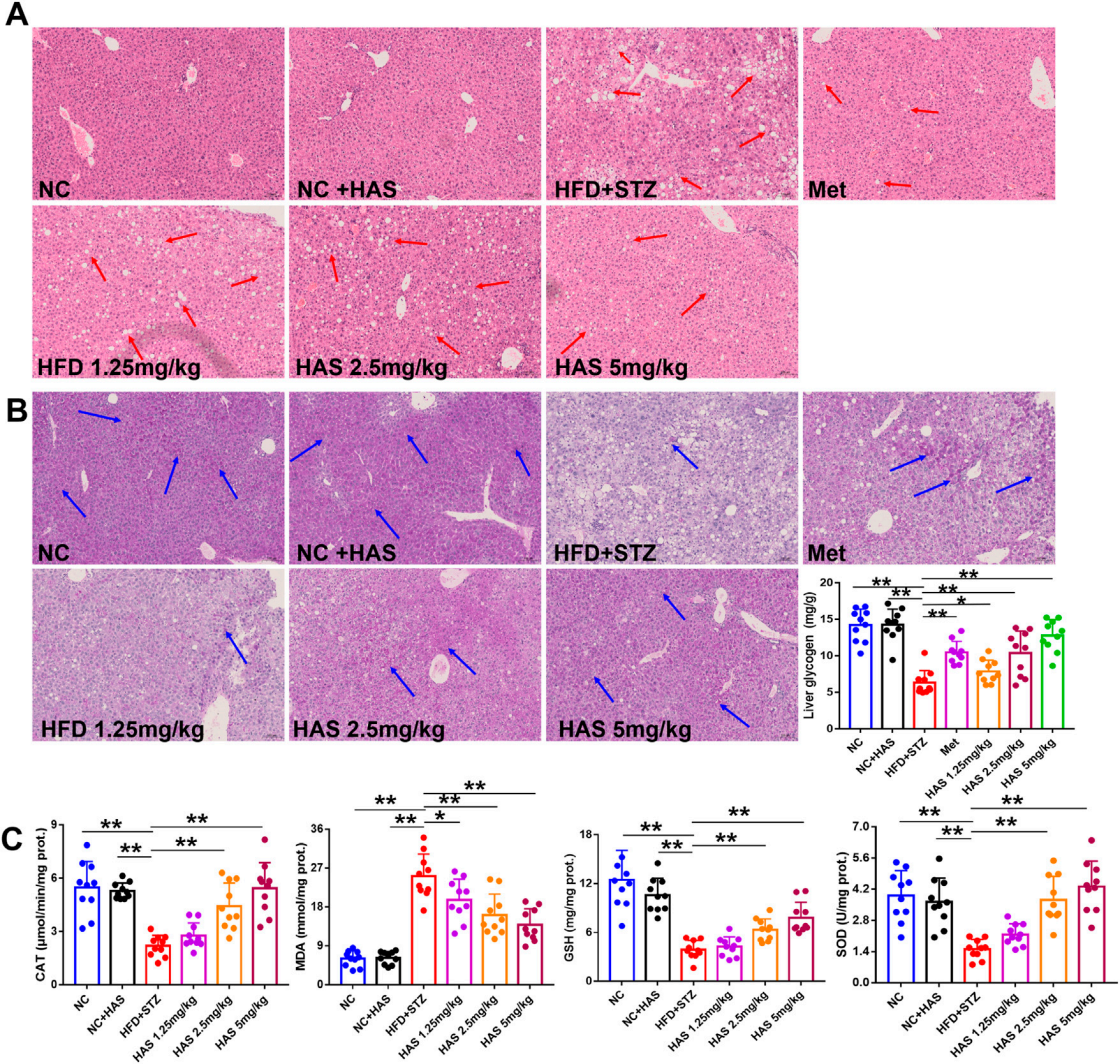
Effects of HAS on histopathology, oxidative stress, and liver glycogen of T2DM mice. Liver sections were stained with HE **(A)** and PAS **(B)** to determine the histopathological changes and liver glycogen content, respectively, in HFD + STZ mice. **(C)** Effects of HAS on oxidative stress in liver tissues of T2DM mice. The doses of 2.5, 5.0, and 10 mg/kg indicate low, middle, and high doses of HAS, and the dose of Met was 150 mg/kg. Data are expressed as mean ± SD (*n* = 10), **p < 0.01 vs. HFD + STZ group.

As demonstrated in Figure 3A, liver cells of normal mice were neatly arranged and compact, with similar cell size and morphology throughout the tissue, clear nuclear contour, and no pathological damage. However, after treatment with HFD + STZ, the liver of T2DM mice showed obvious steatosis, and many vacuoles of different sizes were scattered in the liver cell cytoplasm. The volume of liver cells increased, the cell shape was irregular, and the cytoplasm was fused. Interestingly, both HAS and Met treatment reduced the pathological changes in liver tissues, as the cells in liver tissue from treated mice appeared closely arranged, and vacuoles were significantly reduced.

Oxidative stress is generally believed to be involved in the pathogenesis of diabetes (Kaneto et al., 2007). In this study, the levels of CAT, MDA, GSH, and SOD in the liver were determined. HFD + STZ treatment significantly reduced the levels of CAT, GSH, and SOD, and significantly increased MDA, indicating high levels of oxidative stress in the liver of T2DM mice; importantly, our results showed that HAS treatment reduced oxidative stress in the liver of T2DM mice compared to mice without HAS treatment (Figure 3C).

### HAS increases hepatic glycogen storage in T2DM mice

Glucose metabolism disorder is the most important characteristic of T2DM, and liver glycogen plays an important role in regulating glucose metabolism. PAS staining was used to visualize liver glycogen, and it was found that glycogen-positive staining in the liver of mice was significantly reduced after HFD and STZ treatment, and that glycogen content in the liver gradually recovered after HAS treatment (Figure 3B). Similar results were obtained by quantitative assessment of liver glycogen content. HAS significantly increased liver glycogen content at all doses tested, suggesting that the hypoglycemic effect of HAS on T2DM mice was related to the increase in liver glycogen content (Figure 3B). Similar results were observed with the positive control treatment (Met).

### HAS increases glucose uptake in HepG2 cells induced by glucosamine

**FIGURE 4:**
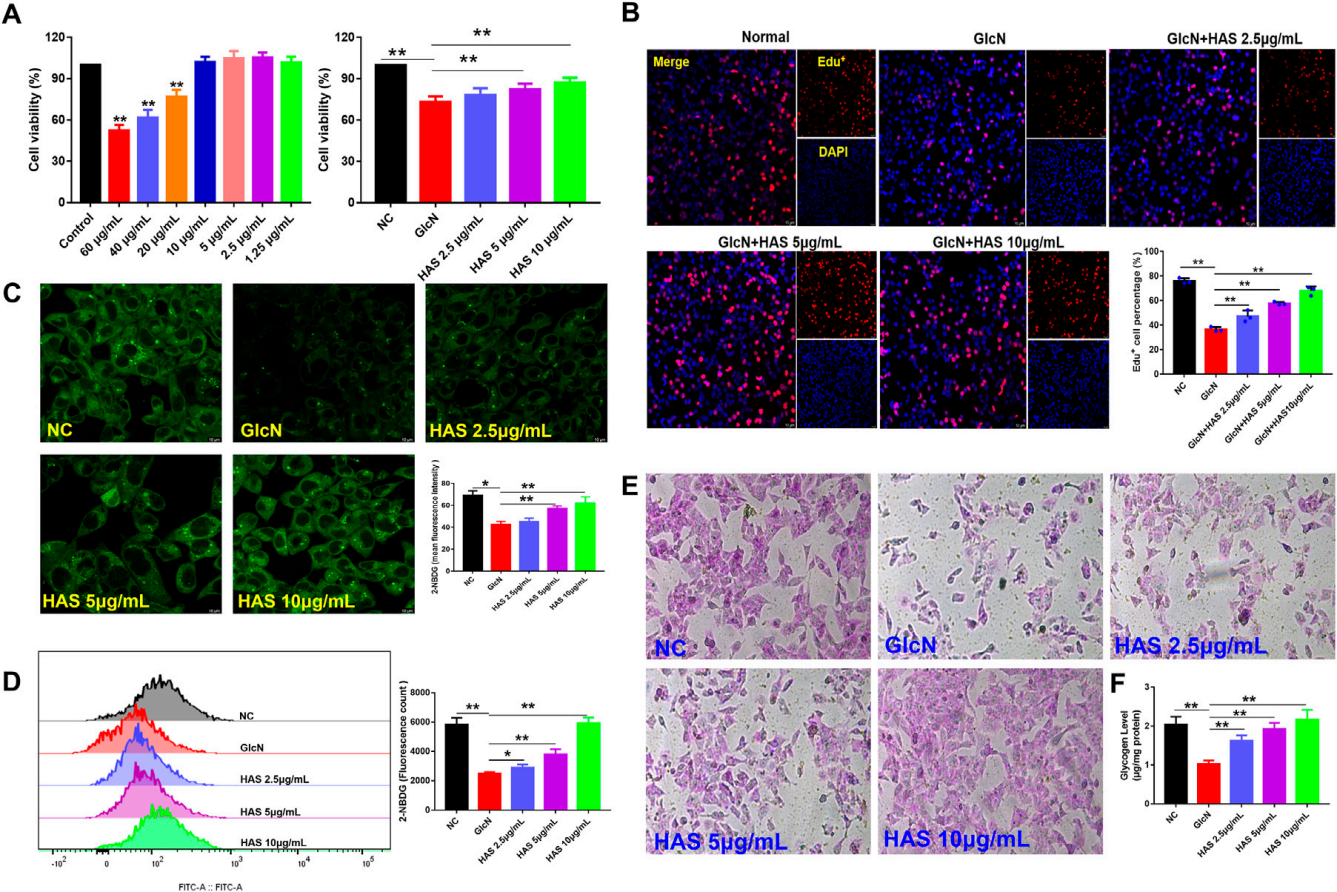
Effects of HAS on cell viability and glycogen content of GlcN-induced HepG2 cells. **(A,B)** HAS increased the viability of GlcN-induced HepG2 cells. **(C,D)** HAS increased the glucose uptake of GlcN-induced HepG2 cells, as visualized using the fluorescent glucose analogue 2-NBDG. **(E,F)** HAS increased the glycogen content in GlcN-induced HepG2 cells. Data are expressed as mean ± SD (*n* = 3), *p < 0.05, **p < 0.01 vs. GlcN-induced HepG2 cells.

The viability of HepG2 cells after HAS treatment was evaluated by CCK-8 assay (Figure 4A). HAS had no obvious inhibitory effect on normal HepG2 cells at doses under 20 μg/ml and HAS treatment at doses of 2.5–10 μg/ml increased the viability of GlcN-induced HepG2 cells (Figure 4A). Furthermore, EdU staining also demonstrated that HAS reduced the inhibitory effect of GlcN on the proliferation of HepG2 cells (Figure 4B). *In vivo*, we observed that HAS increased glycogen content in the liver of T2DM mice (Figure 4B). We also detected glycogen content in GlcN-induced HepG2 cells. GlcN can induce insulin resistance in HepG2 cells and reduce the efficiency of glucose uptake and utilization in hepatocytes (Xiao et al., 2011; Sun et al., 2020). The fluorescent glucose analogue 2-NBDG is used to visualize glucose uptake by living cells and is often used to evaluate the glucose uptake capacity of cells. As shown in Figures 4C,D, confocal laser microscopy and flow cytometry results demonstrated that GlcN-induced HepG2 cell uptake of 2-NBDG was significantly reduced compared with the NC cells, but HAS treatment reduced GlcN-induced inhibition of glucose uptake in HepG2 cells. Additionally, our results indicate that HAS increased PAS-positive staining and cell glycogen content in GlcN-induced HepG2 cells (Figures 4E,F).

### HAS increases hepatocyte glycogen accumulation *via* the PI3K/Akt/GSK-3β pathway

**FIGURE 5:**
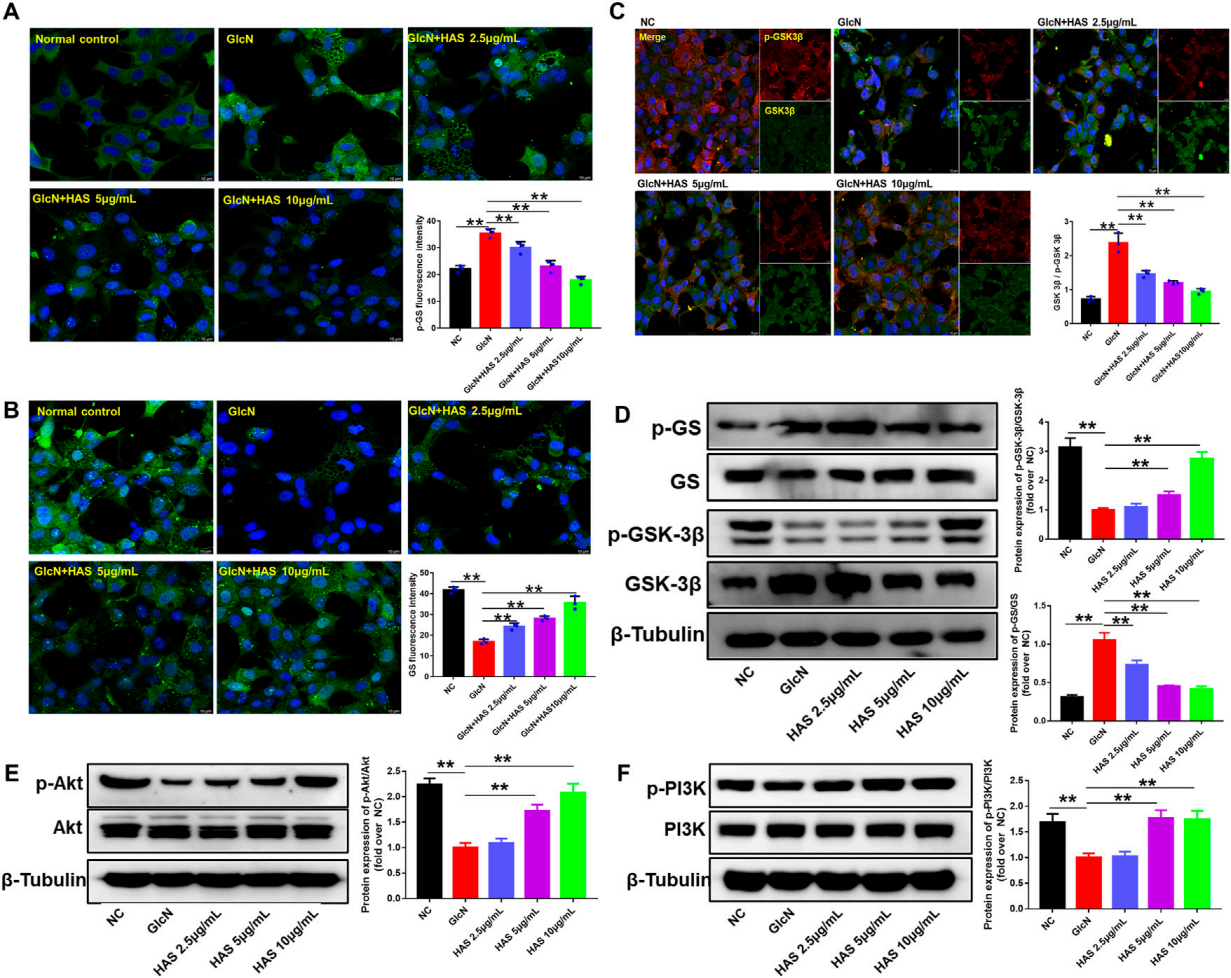
Effects of HAS on expression of PI3K/Akt/GSK-3β signaling pathway proteins in GlcN-induced HepG2 cells. **(A,B)** HAS decreased the phosphorylation of GS in GlcN-induced HepG2 cells. **(C)** HAS increased the expression of p-GSK-3β/GSK-3β in GlcN-induced HepG2 cells. **(D)** Western blotting assays of p-GS, GS, p-GSK-3β, and GSK-3β in GlcN-induced HepG2 cells. **(E,F)** HAS increased the phosphorylation of PI3K and Akt in GlcN-induced HepG2 cells. Data are expressed as mean ± SD (*n* = 3), *p < 0.05, **p < 0.01 vs. GlcN-induced HepG2 cells.

To investigate the possible mechanisms of HAS antidiabetic bioactivity, we studied the effect of HAS treatment on the PI3K/Akt/GSK-3β pathway in GlcN-induced HepG2 cells. As shown in Figure 5, results of western blotting and immunofluorescence staining indicate that GlcN stimulation significantly reduced the phosphorylation of PI3K, Akt, and GSK-3β in HepG2 cells, whereas GlcN stimulation increased the phosphorylation of GS. Interestingly, HAS effectively up-regulated the phosphorylation of PI3K, Akt, and GSK-3β in GlcN-induced HepG2 cells, whereas HAS down-regulated the phosphorylation of GS.

**FIGURE 6:**
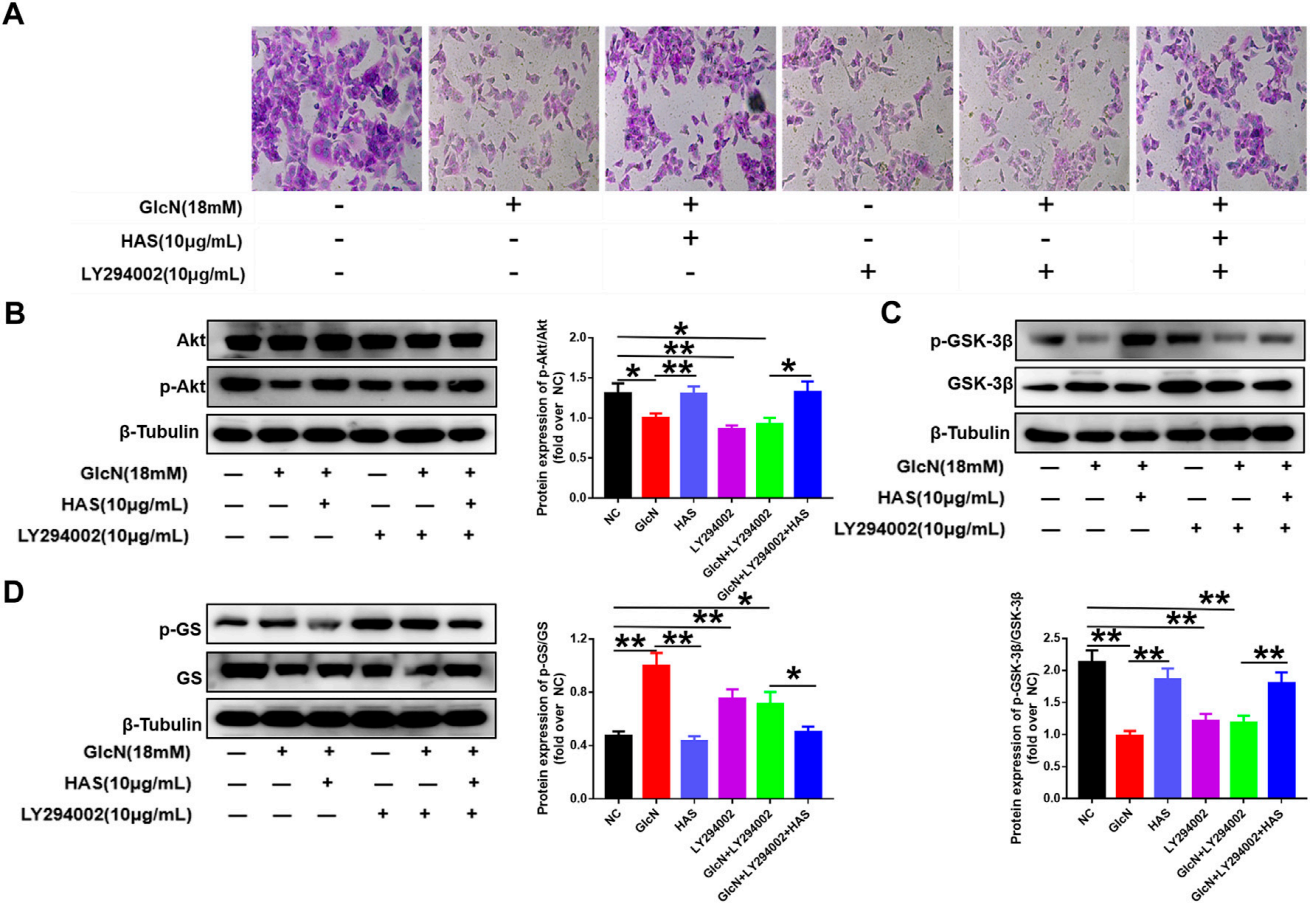
Effects of the PI3K inhibitor LY294002 on glycogen content and PI3K/Akt/GSK-3β signaling in GlcN-induced HepG2 cells. **(A)** The effect of HAS in promoting glycogen increase in GlcN-induced HepG2 cells was inhibited by the PI3K inhibitor LY294002. **(B–D)** The activation of the PI3K/Akt/GSK-3β pathway by HAS in GlcN-induced HepG2 cells was inhibited by the PI3K inhibitor LY294002. Data are expressed as mean ± SD (*n* = 3), *p < 0.05, **p < 0.01 vs. GlcN-induced HepG2 cells.

Furthermore, the PI3K/Akt pathway inhibitor LY294002 was used to confirm the involvement of the PI3K/Akt/GSK-3β pathway in the effects of HAS on T2DM. As shown in Figure 6, GlcN or LY294002 treatment reduced glycogen accumulation in HepG2 cells, and the addition of LY294002 further inhibited glycogen accumulation in GlcN-induced HepG2 cells. Furthermore, blocking PI3K with LY294002 suppressed HAS-mediated up-regulation of PI3K, Akt, and GSK-3β phosphorylation in GlcN-induced HepG2 cells, as well as suppressed HAS-mediated down-regulation of GS phosphorylation.

## Conclusion

T2DM is a common chronic endocrine and glucometabolic disease, accounting for more than 90% of all cases of diabetes. Due to changes in dietary habits, T2DM incidence is increasing year by year worldwide, especially in developed and developing countries, which show trends toward epidemic levels of the disease (Sun et al., 2018; Kim et al., 2019). Diabetes has become one of the most prevalent non-infectious diseases threatening human health and life and is ranked third after cardiovascular disease and cancer. T2DM is primarily caused by the relative insufficiency of insulin secretion or the insulin resistance of various organs. Characterized by a significantly increased blood glucose level, T2DM can lead to damage to multiple organ systems in the body. T2DM patients often endure a series of complications caused by hyperglycemia. Currently, metformin, α-glucosidase inhibitors, sulfonylureas, and DPP-4 inhibitors are clinical drugs commonly used for treating T2DM. However, all the currently available drugs come with potentially serious side effects, such as renal impairment, gastrointestinal disorders, and hypoglycemia. Therefore, finding new antidiabetic drugs is necessary for the prevention and clinical treatment of T2DM. In our study, we investigated the antidiabetic effects of HAS against T2DM and explored the potential related molecular mechanisms. Previous studies have reported that zanthoxylamides extracted from *Z. bungeanum* have potential antidiabetic effects in mice *via* activation of the AMPK/PI3K/Akt signaling pathway (Xu et al., 2022; Zhang et al., 2022). In our study, we found that HAS, which is the most important zanthoxylamide in *Z. bungeanum*, has potential antidiabetic effects against T2DM based on investigation using a high-fat-fed and streptozotocin-induced T2DM mouse model.

Hepatic glycogen synthesis and storage is an important biochemical process to ensure the survival of the human body. Insulin resistance induced by T2DM can inhibit hepatic glycogen synthesis and lead to hyperglycemia (Liu et al., 2015; Flannick et al., 2016). GS is a key enzyme in the process of glycogen synthesis, and its activity is regulated by cycles of phosphorylation (inactive) and dephosphorylation (active). It is known that the enzyme activity of GS is negatively regulated by its phosphorylation, which is primarily regulated by GSK-3, a serine/threonine protein kinase with two isomers, namely GSK-3α and GSK-3β. GSK-3 inhibits glycogen synthesis by catalyzing GS phosphorylation to inactivate it. Furthermore, previous studies have shown that hepatic insulin resistance is usually related to the inhibition of the PI3K/Akt pathway (Whiteman et al., 2002; Yan et al., 2016; Jiang et al., 2021). Activation of PI3K/Akt controls glucose homeostasis through a variety of pathways, including *via* increasing glucose uptake in muscle and adipose tissue (Sano et al., 2007), promoting glycogenesis in liver and muscle (Cross et al., 1995; Irimia et al., 2010), and inhibiting hepatic gluconeogenesis (Puigserver et al., 2003; Matsumoto et al., 2007). The PI3K/Akt pathway is the main upstream regulatory link with GSK-3 and is widely recognized as a crucial step in gluconeogenesis and glycogen synthesis in T2DM (Wang et al., 2014). Generally, activated Akt phosphorylates and inactivates the downstream target of GSK-3 in response to insulin stimulation. In this way, the negative regulatory effects of GSK-3β on GS are removed, and the activity of GS is increased, thus promoting glycogen synthesis. In our study, we confirmed that HAS increases the phosphorylation levels of PI3K and Akt and induces the phosphorylation and inactivation of GSK-3β, thereby removing its inhibitory effect on its target enzyme (GS) and promoting liver glycogen synthesis. As confirmation of PI3K/Akt involvement, the PI3K inhibitor LY294002 blocked PI3K/Akt signaling, which led to reduced accumulation of liver glycogen in insulin-resistant cell models treated with HAS.

In conclusion, our results suggest that HAS reduces blood glucose in the T2DM mouse model by regulating glycogen metabolism through a mechanism involving the regulation of the PI3K/Akt/GSK3β/GS signaling pathway. These data support the need for further exploration of HAS as a safe natural supplement or additional therapeutic option in the prevention or treatment of T2DM.

## Data Availability

The original contributions presented in the study are included in the article/Supplementary Materials, further inquiries can be directed to the corresponding authors.

## Supplementary material

Supplementary Figure S1
**(A)** HAS reduced the water intake of HFD + STZ mice. **(B)** HAS reduced the food intake of HFD + STZ mice. **(C)** Fasting blood glucose (FBG) of T2DM mice per week.
