# Supplementary figures and images for: Hydroxy-α-sanshool isolated from Zanthoxylum bungeanum Maxim. has antidiabetic effects on high-fat-fed and streptozotocin-treated mice via increasing glycogen synthesis by regulation of PI3K/Akt/GSK-3β/GS signaling
Source: Front Pharmacol. 2022 Dec 13;13:1089558. doi: 10.3389/fphar.2022.1089558 (PMC9792598; doi:10.3389/fphar.2022.1089558)

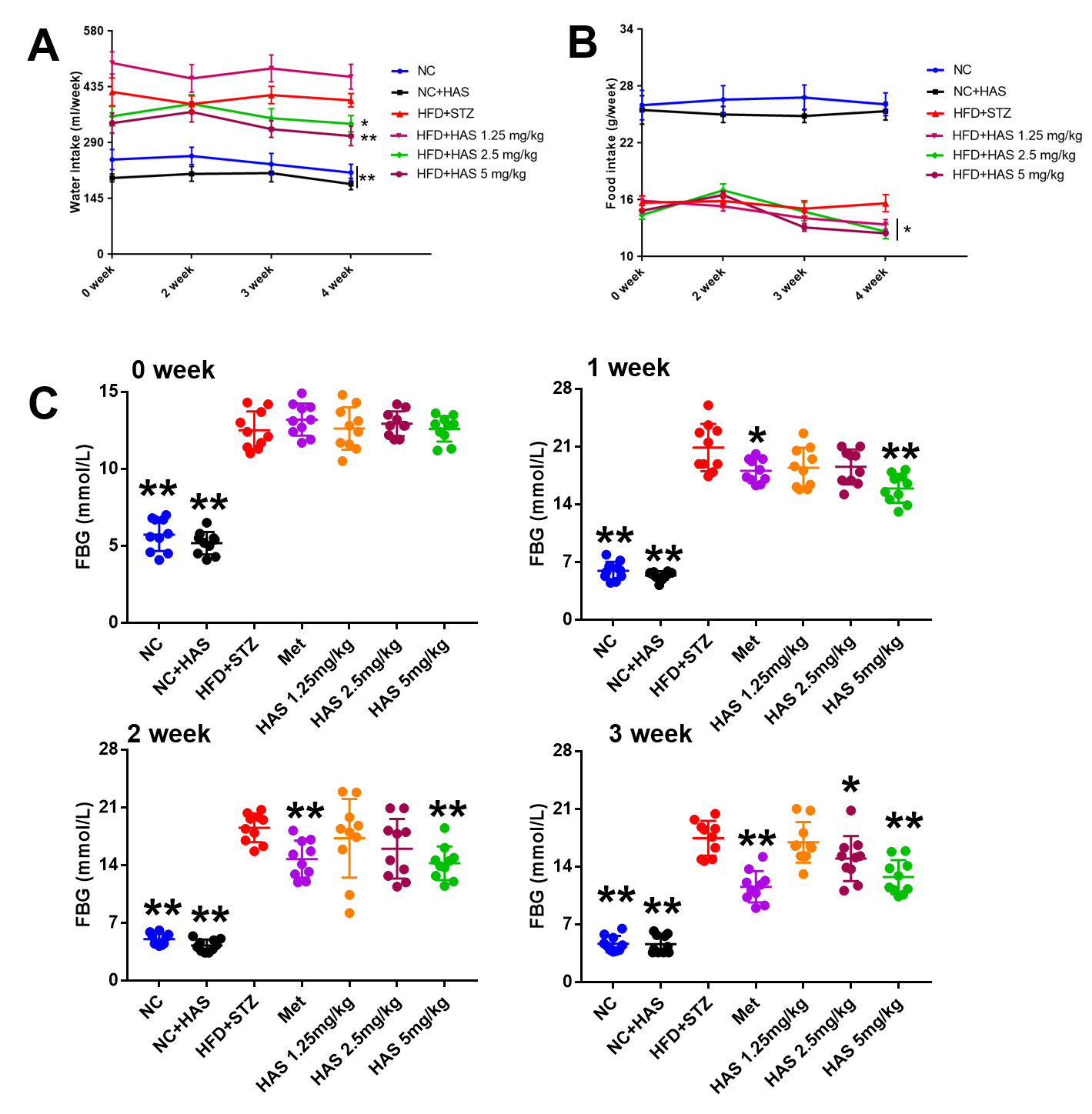

Supplement: Supplementary file 1 [file Image1.tif]
